# On‐Chip Biomimetic Intelligence: Integrating Adaptive Molecular Sensing With Longitudinal Proteomic Profiling for Advanced Disease Diagnostics

**DOI:** 10.1002/cbdv.71908

**Published:** 2026-11-05

**Authors:** Bakr Ahmed Taha, Ali J. Addie, Khansaa Basem Fadhil, Adawiya J. Haider, Ahmed Jamal Jasim, Norhana Arsad

**Affiliations:** ^1^ Photonics Technology Lab, Department of Electrical, Electronic and Systems Engineering, Faculty of Engineering and Built Environment Universiti Kebangsaan Malaysia UKM Bangi Selangor Malaysia; ^2^ Al‐imam University College Balad Iraq; ^3^ Center of Industrial Applications and Materials Technology Scientific Research Commission Baghdad Iraq; ^4^ Department of Technical Medical Laboratory Northern Technical University Mosul Iraq; ^5^ Applied Sciences Department/Laser Science and Technology Branch University of Technology Baghdad Iraq; ^6^ University of Technology Baghdad Iraq; ^7^ Department of Engineering, Faculty of Advanced Technology and Multidiscipline Universitas Airlangga Surabaya Indonesia

**Keywords:** biocompatible nanomaterials, biomimetic intelligence, digital twins, molecular recognition, on‐chip

## Abstract

Biological, molecular, and functional states evolve across multiple timescales, limiting the utility of conventional diagnostics based on isolated measurements. This Perspective introduces on‐chip biomimetic intelligence as an integrated approach combining biomimetic molecular recognition and signal transduction with dynamic sensing, adaptive local computation, and longitudinal interpretation of molecular changes. It focuses on multiplexed molecular‐affinity measurements of selected disease‐associated protein panels, coupled with event‐driven signal processing to reduce redundant data generation. Where appropriate, sensing outputs may inform patient‐specific digital twin models and dynamically update PBPK or PK–PD models. Rather than treating intelligent biosensors, neuromorphic biosensing, edge AI, and digital twins as isolated technologies, it integrates their functions and describes architectural and functional requirements for chip‐level biomimetic intelligence. Implementation requires regenerable molecular–recognition interfaces, reliable molecular‐to‐signal transduction, and computational methods capable of estimating protein states and temporal trajectories. Causal validation and constrained closed‐loop control may support clinically relevant decision‐making when sufficient evidence is available. However, complete architecture has not yet been demonstrated as a single clinically validated system. Its realization requires advances in sensing stability, biocompatibility, antifouling performance, calibration, drift control, and clinical validation. Thus, biosensing shifts from static biomarker detection to longitudinal, adaptive, and clinically interpretable monitoring of changes in biological state.

## Graphical abstract

On‐chip biomimetic intelligence integrates molecular recognition, dynamic sensing, adaptive computation, and longitudinal molecular‐state interpretation. Multiplexed protein monitoring is coupled with event‐efficient signal processing and, where appropriate, patient‐specific digital twins. The framework advances biosensing from static detection toward adaptive, longitudinal, and clinically interpretable molecular‐state estimation, while emphasizing uncertainty, causal validation, calibration stability, and staged clinical translation.

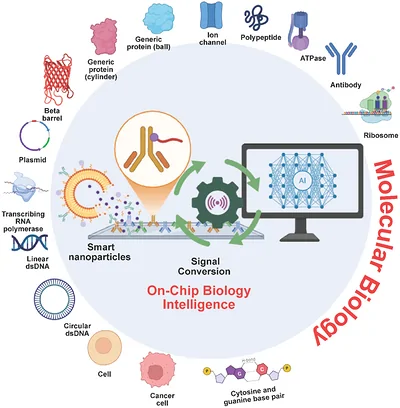

**Abbreviations**
PBPK: physiologically based pharmacokinetic
PK–PD: pharmacokinetic–pharmacodynamic

## 1 Introduction

Living systems have complex molecular pathways that are constantly changing, proving that traditional analytical methods are inadequate. Modern techniques require integrated platforms that go beyond static measurements to enable real‐time, adaptive interpretation of cellular activities. On‐chip systems can integrate multiple sensing modalities and adaptive decision‐making capabilities due to smart nanoscale and microscale sensors [1]. Bio‐inspired sensing, imaging, and nanoscale biomedical platforms have expanded molecular diagnostics across recognition, classification, and targeted intervention [2, 3, 4, 5, 6]. In the human proteome, protein quantities, interactions, and modifications evolve with normal and pathological physiology. Nanoscale optical and bioelectronic approaches increasingly seek to capture and interpret such molecular and physiological signals [7, 8, 9]. Integrating artificial intelligence (AI) with molecular biology provides computational tools for interpreting complex molecular datasets and identifying relationships that are difficult to resolve using conventional analysis alone. Computational learning and data‐analysis strategies have consequently been explored across clinical risk prediction, biomedical image interpretation, and nanotechnology‐enabled diagnostics [10, 11, 12]. Machine learning, particularly deep neural networks and supervised learning, has recently emerged as a crucial component in elucidating structure within extensive genomics and multi‐omics datasets, as well as linking molecular variation to phenotype and mechanism [13]. Advanced computational analysis can support personalized diagnosis and precision medicine. AI methods and nanophotonic or electrochemical platforms are being explored across biomedical sensing, signal interpretation, and decision support [14, 15, 16, 17]. These developments allow integrated sensors to couple molecular readout with local pattern recognition and adaptive decision‐making, creating a route to resolving spatiotemporal molecular and physiological dynamics [18, 19, 20, 21]. In the present framework, the primary on‐chip route envisaged for proteome profiling is multiplexed affinity‐based sensing using arrays of aptamers or other engineered recognition elements integrated with microfluidic sampling and electrical or optical transduction. This architecture is particularly compatible with repeated low‐volume measurements of selected protein panels rather than exhaustive whole‐proteome characterization. Nanopore and other single‐molecule technologies provide complementary routes toward label‐free, higher‐resolution protein identification [22, 23], whereas mass spectrometry is considered primarily an orthogonal method for reference profiling and validation rather than an integral on‐chip component. In this Perspective, proteome profiling refers primarily to longitudinal monitoring of a selected panel of disease‐relevant proteins rather than comprehensive, unbiased analysis of the entire proteome. The selected proteins are intended to provide temporally resolved molecular signatures that can be repeatedly measured and integrated with adaptive inference and disease‐state modeling. Broad proteomic approaches may instead serve upstream for biomarker discovery, panel selection, and independent validation [24, 25].

Multiplexing alone does not establish a strict boundary between biosensing and proteomic profiling. For the purposes of this Perspective, we operationally distinguish conventional multiplexed biosensing, which measures a limited predefined set of diagnostic targets, from longitudinal proteomic profiling, which interrogates a sufficiently broad protein panel to characterize multivariate biological states and their temporal evolution. As a practical criterion, we consider approximately ≥ 50 simultaneously or serially monitored proteins spanning multiple pathways to represent the lower high‐plex regime for targeted proteomic profiling, while recognizing that this is not a universal proteomics threshold. Published affinity‐based proteomic studies commonly interrogate tens to hundreds or more proteins, including 88‐, 593‐, and 813‐protein datasets [26, 27]. Importantly, a smaller subset subsequently selected for clinical prediction should be described as a biomarker panel rather than as a proteome profile.

With the molecular scope defined above, wearable biosensors and point‐of‐care platforms provide a practical route for repeated, minimally invasive measurement of clinically relevant biomarkers [28, 29, 30, 31, 32, 33]. In physiological contexts, these devices must distinguish target‐dependent signals from matrix interference and background variability. Biomimetic strategies address this requirement by reproducing selected principles of biological recognition, amplification, and filtering [34, 35, 36]. Biomimetic materials can reproduce selected biological interface properties in biosensor devices. Cell‐membrane‐like nanostructures provide surface chemistries that can stabilize interactions with target biomolecules; adhesion molecules on membrane‐coated nanoparticles, for example, influence biomolecular interactions and therapeutic targeting [37]. Together, these approaches support more selective biomarker measurement and repeated disease monitoring. Figure 1. Digital‐twin–synchronized on‐chip inference based on multiscale biomimetic intelligence.

**FIGURE 1:**
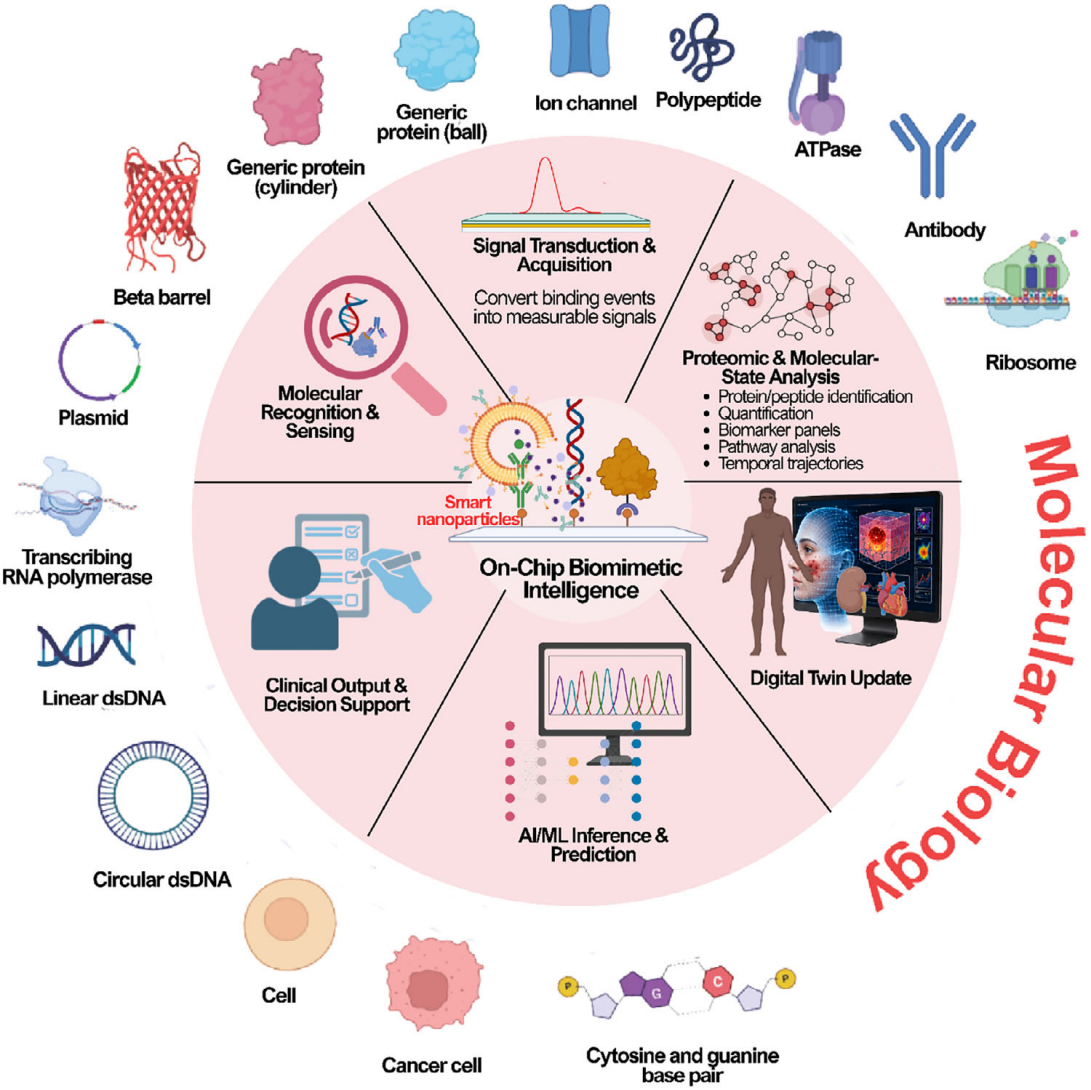
Prospective multiscale architecture of biomimetic intelligence from molecular recognition and sensing to on‐chip signal processing, proteomic and molecular‐state evaluation, digital‐twin enhancement, and AI‐driven clinical deduction. The molecular, genetic, and cellular components demonstrate the biological fundamentals of sensing and information processing, while the unified structure demonstrates the transformation of molecular events into personalized actionable outputs for patients through digital‐twin synchronization and adaptive feedback.

DNA‐functionalized nanoparticle platforms illustrate one implementation of biomimetic recognition coupled with nanoscale signal transduction [38]. Such platforms illustrate how biomimetic recognition can be integrated with responsive materials for disease characterization and monitoring. In this Perspective, biomimetic intelligence is defined as a systematic framework for decoding and manipulating dynamic biological information. Here, on‐chip biomimetic intelligence refers to an integrated sensing–inference framework in which biomimetic molecular recognition and transduction are coupled with local adaptive computation and dynamic molecular‐state interpretation. It is broader than an intelligent biosensor, where AI primarily assists in signal interpretation, classification, or prediction, and differs from neuromorphic biosensing, which specifically implements brain‐inspired or event‐driven computation in hardware. Edge‐AI biosensors are primarily distinguished by performing inference locally at or near the sensor rather than through remote computation, whereas digital‐twin diagnostics center on patient‐specific computational models continuously informed by clinical or molecular data. The framework proposed here links these otherwise distinct functions through molecular sensing, adaptive local inference, longitudinal molecular/proteomic profiling, and, where appropriate, synchronization with predictive digital‐twin models. In this context, “on‐chip” refers primarily to the miniaturized sensing and local inference interface and does not imply that the complete digital twin or proteomic model must physically reside on a single chip.

Within this framework, a system qualifies as on‐chip biomimetic intelligence when three core features are integrated: (i) a biomimetic sensing interface that reproduces a biological principle of molecular recognition, transduction, amplification, or filtering; (ii) dynamic sensing that captures changes in molecular state rather than only a static endpoint; and (iii) adaptive local computation or inference that modifies its interpretation according to incoming biological information. Thus, biomimetic materials or AI‐assisted signal analysis alone are insufficient to constitute biomimetic intelligence. Features such as self‐healing, proteomic integration, digital‐twin synchronization, and closed‐loop therapeutic control can extend the framework but are not mandatory criteria.

Here, adaptation is considered a system‐level property that may operate at several layers. At the sensing interface, reversible or actively regenerated recognition elements can restore receptor availability as analyte concentrations change [39]. At the acquisition level, event‐driven sampling can modify measurement frequency according to signal dynamics, while adaptive calibration can update baseline or drift corrections using reference channels. At the computational level, local models can update parameters, representations, or decision boundaries in response to new molecular measurements [21]. Within the present framework, adaptive local inference is the minimum defining requirement; adaptive recognition, calibration, and sampling are additional implementation strategies. Accordingly, conventional sensing followed only by fixed or remote downstream data analysis is not considered an adaptive on‐chip sensor. To capture spatiotemporal biological organization, we envisage a nonlinear latent state‐space framework in which the high‐dimensional molecular observation vector ($y_{t}$) is mapped to a lower‐dimensional latent biological state ($z_{t}$):

$$zt=E\phi \left(y_{t}\right)$$

whose temporal evolution is represented as follows:

$$\left[zt+1=F\theta \left(z_{t},u_{t}\right)+w_{t}\right]$$

where ($E_{\phi }$) denotes the learned encoder, ($F_{\theta }$) describes the nonlinear state dynamics, ($u_{t}$) represents measured perturbations or interventions, and ($w_{t}$) represents process uncertainty. Reduced coordinates and governing dynamics may be learned using autoencoder‐assisted sparse dynamical modelling or Koopman‐based representations [40, 41]. Within this state space, stable fixed points satisfying as follows:

$$\left[z=F_{\theta }\;\left(z,u^{*}\right)\right]$$

or, recurrent trajectories provide operational representations of attractor‐like physiological states. Time‐series causal inference can subsequently identify candidate directed molecular dependencies [42], which require mechanistic or interventional validation before being interpreted as causal, while network controllability analysis can prioritize candidate control nodes for subsequent perturbational validation [43]. Adaptation is envisaged through online updating of ($E_{\phi }),(F_{\theta }$), or their parameters as new sensing data become available. This formulation represents a prospective computational implementation of the proposed framework rather than a single prescribed algorithm.

The proposed architecture should be distinguished from its individually demonstrated components. Experimental platforms have already established on‐chip affinity‐based protein sensing with time‐resolved readout [44], biosensor‐integrated neuromorphic hardware capable of on‐chip learning and classification [21], multi‐sensor organ‐on‐chip systems for automated continual physiological monitoring [45], and biochips for multiplexed immune‐profiling [46]. Patient‐specific digital twins have likewise been implemented computationally using longitudinal clinical and pharmacological data [47]. However, these capabilities currently exist largely as separate technological modules. Their integration into a single architecture that combines longitudinal high‐plex protein‐panel sensing, adaptive local inference, dynamical‐state and causal modelling, digital‐twin synchronization, and autonomous feedback remains conceptual. Accordingly, the present Perspective proposes a system‐level convergence pathway rather than claiming that the complete biomimetic‐intelligence architecture has already been experimentally realized.

## 2 Adaptive Transduction of Sensing Technologies

Transduction is the process by which a biosensor converts a biological or chemical signal (analyte binding, reaction, or molecular interaction) into a quantifiable and interpretable output signal. The biological recognition event generates a measurable signal that corresponds to the quantity or concentration of the analyte [48]. Transduction techniques include electrochemical, optical, plasmonic, fluorescence, and terahertz‐based platforms, each with unique advantages depending on the application context. Furthermore, transduction mechanisms that convert molecular data into signals detectable by electrical or optical techniques are crucial for biosensor technologies. Thus, achieving this complex process requires attention to several key factors to maintain biochemical fidelity during transmission, such as stable interfaces, selectivity, and compatibility with the body [48, 49, 50].

Molecular recognition in aqueous biofluids can be achieved by combining the sequence‐specific binding of DNA aptamers with electrochemical transduction, in which target binding is converted into a measurable electrical signal. Interface stability and analytical selectivity are key determinants of biosensor performance, governing signal drift, nonspecific adsorption, and operational lifetime. Bioelectrode materials, surface functionalization, and fabrication directly influence analytical sensitivity, selectivity, and operational stability [49, 51]. Biorecognition components must be immobilized on electrode surfaces in ways that enhance signal transduction and minimize interference from nontarget molecules. Graphene and carbon‐nanotube composites are widely investigated as flexible biosensor materials because of their electrical conductivity and mechanical properties. According to Gazzato and Frasconi, carbon nanotube composites can improve selectivity and response time in electrochemical biosensors due to their outstanding conductivity and mechanical flexibility. For flexible biosensing applications, signal transducers must combine electrical performance with mechanical compatibility [51]. ZnO nanostructures provide high‐surface‐area interfaces suitable for biomolecular immobilization and electrochemical transduction. For example, nanostructured ZnO electrodes have enabled affinity‐based detection of cardiac troponin T in human serum using low sample volumes, illustrating their applicability to biomolecular sensing in complex matrices [52]. MOF‐based nanozymes combine tunable surface chemistry with catalytic activity, providing a route to signal amplification and improved analytical performance in complex matrices [53]. Label‐free, high‐resolution analysis of individual molecules using translocation current modulation is possible with nanopore‐based systems (Figure 2). Organic electrochemical transistors convert ionic interactions in biological media into amplified electrical signals through conducting‐polymer channels, as shown in Figure 2a. Enzymes with metal or carbon electrodes facilitate mediated electron transfer at redox‐active biointerfaces, as shown in Figure 2b. Engineered whole‐cell biosensors produce measurable outputs, such as GFP detected through optical fibers, to report on molecular or metabolic activity in Figure 2c. Synthetic membranes containing protein nanopores permit DNA translocation, producing characteristic ionic‐current blockade pulses for molecular identification, as shown in Figure 2d. Finally, enhanced biosensing technologies that leverage safe transduction processes for living systems are needed for environmental monitoring and medical diagnostics. By using new sensor architectures and functional materials, biosensor systems can be durable, selective, and efficient while maintaining signal integrity. Through conducting polymers, organic electrochemical transistors amplify ionic activity into electrical signals for sensitive, tissue‐compatible bioelectronic sensing. Engineered whole‐cell biosensors and redox‐active bio‐interfaces can quantify electrochemical or optical signals from analyte detection or metabolic activities using electron transfer and cellular signal processing.

**FIGURE 2:**
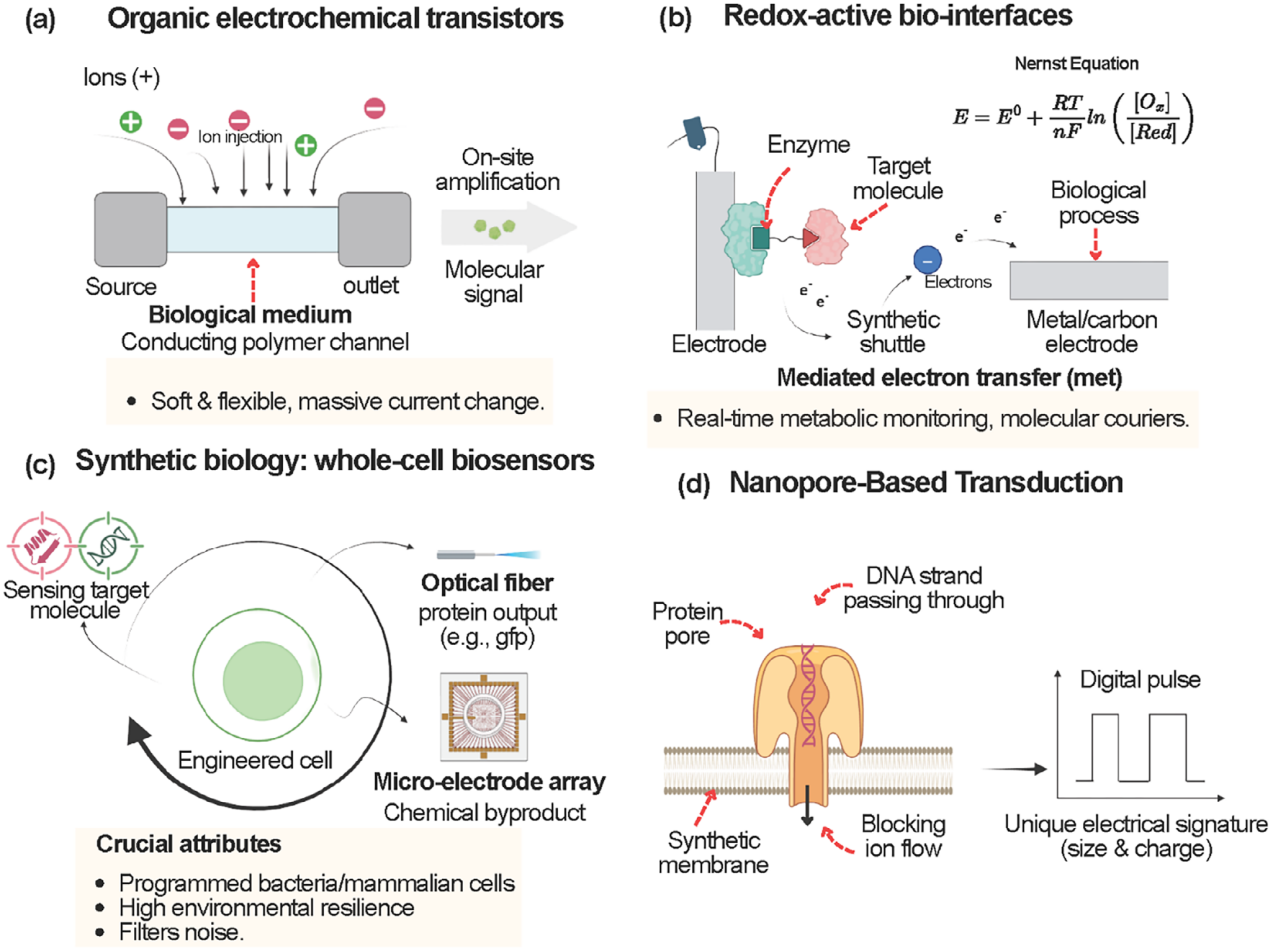
An overview of synthetic and bioelectronic sensing systems, showing (a) ionic signal transduction using organic electrochemical transistors, (b) redox‐active electrochemical interfaces, (c) engineered whole‐cell biosensors, and (d) nanopore‐based molecular detection.

## 3 Aspects of On‐Chip Biomimetic Intelligence

### 3.1 Molecular Recognition and Capture

Two examples of how advances in on‐chip biological intelligence have enabled new ways to discover and capture molecules are aptamers and synthetic receptors. These recognition elements aim to mimic the high affinity and specificity of natural antibodies while avoiding many of the problems associated with them. Lab‐on‐a‐chip and integrated biosensing platforms can favor aptamers and synthetic receptors because of their chemical stability, reusability, and suitability for scalable fabrication. Aptamers and synthetic receptors are becoming increasingly important for developing small diagnostic tools. This section examines their recognition mechanisms and integration into compact diagnostic platforms. Aptamers are short, single‐stranded DNA sequences that can bind to a wide range of targets, including proteins, small molecules, and even whole cells [24, 34]. Aptamers can be immobilized on sensor surfaces while retaining target‐responsive conformational flexibility, making them effective biosensor recognition elements. Their structural adaptability supports high target specificity. The rational design and optimization of aptamers via in vitro selection methods, such as SELEX, enable the creation of highly selective probes tailored for specific sensing applications. Tripathi et al. demonstrated that combining aptamers with nanotechnology can support compact, sensitive, and portable biosensing devices. Nanomaterial integration can enhance signal transduction, while aptamer‐functionalized nanoparticles provide selective biomarker recognition with reduced nonspecific interference. These characteristics support applications in clinical diagnostics, food safety, and environmental monitoring [34]. Synthetic receptors provide engineered alternatives to biological recognition elements and can be designed for selective molecular binding, improved chemical stability, and reproducible fabrication. Computational modeling and predictive design increasingly support the optimization of binding efficiency and reliability. Scalable synthesis can also reduce batch‐to‐batch variability and some stability limitations associated with biological antibodies [34, 35]. This improves reproducibility across fabricated sensing interfaces and supports multiplexed on‐chip detection. Dupin et al. showed that genetically programmable biochips can reproduce complex molecular interactions in confined architectures, supporting high‐throughput analysis [46, 54].

These developments support rapid biomarker monitoring and are particularly relevant to diseases that require sensitive, repeated molecular assessments, including cancer. The integration of aptamers and synthetic receptors with microfluidic devices provides a practical route toward compact on‐chip molecular intelligence. These recognition elements can reduce sample requirements and support improved analytical sensitivity and selectivity. Wang et al. demonstrated a highly sensitive detection of CA125, a clinically significant serum biomarker for ovarian cancer, utilizing an aptamer‐based sensing system [55]. Microfluidic technologies and molecular‐recognition strategies can therefore be integrated to strengthen point‐of‐care diagnostics. In environmental and therapeutic settings, these devices enable real‐time monitoring and rapid analytical results. The on‐chip platforms are efficient and suitable for wider deployment due to their lower sample volume requirements and improved operational efficiency. According to Beatty et al., cyclodextrins can bind organic molecules in water due to the hydroxyl groups on their surfaces. Hydrophobic pockets can be created by synthesizing several water‐soluble cyclophane derivatives containing charged functional groups. For the construction of charged hosts, ions such as sulfonate, carboxylate, phosphonate, ammonium, guanidinium, and pyridinium have been employed. The solubility in water is enhanced by the addition of polyethene glycol tails and other neutral polar functional groups [35]. Figure 3 provides a synopsis of molecular recognition platforms showing the basic detection mechanisms, tailored synthetic binding sites, and analytical methods for selective molecular characterization. Collectively, these recognition elements provide the selective molecular interface required for multiplexed sensing; the next step is to convert that molecular state into a temporally informative signal suitable for local computation.

**FIGURE 3:**
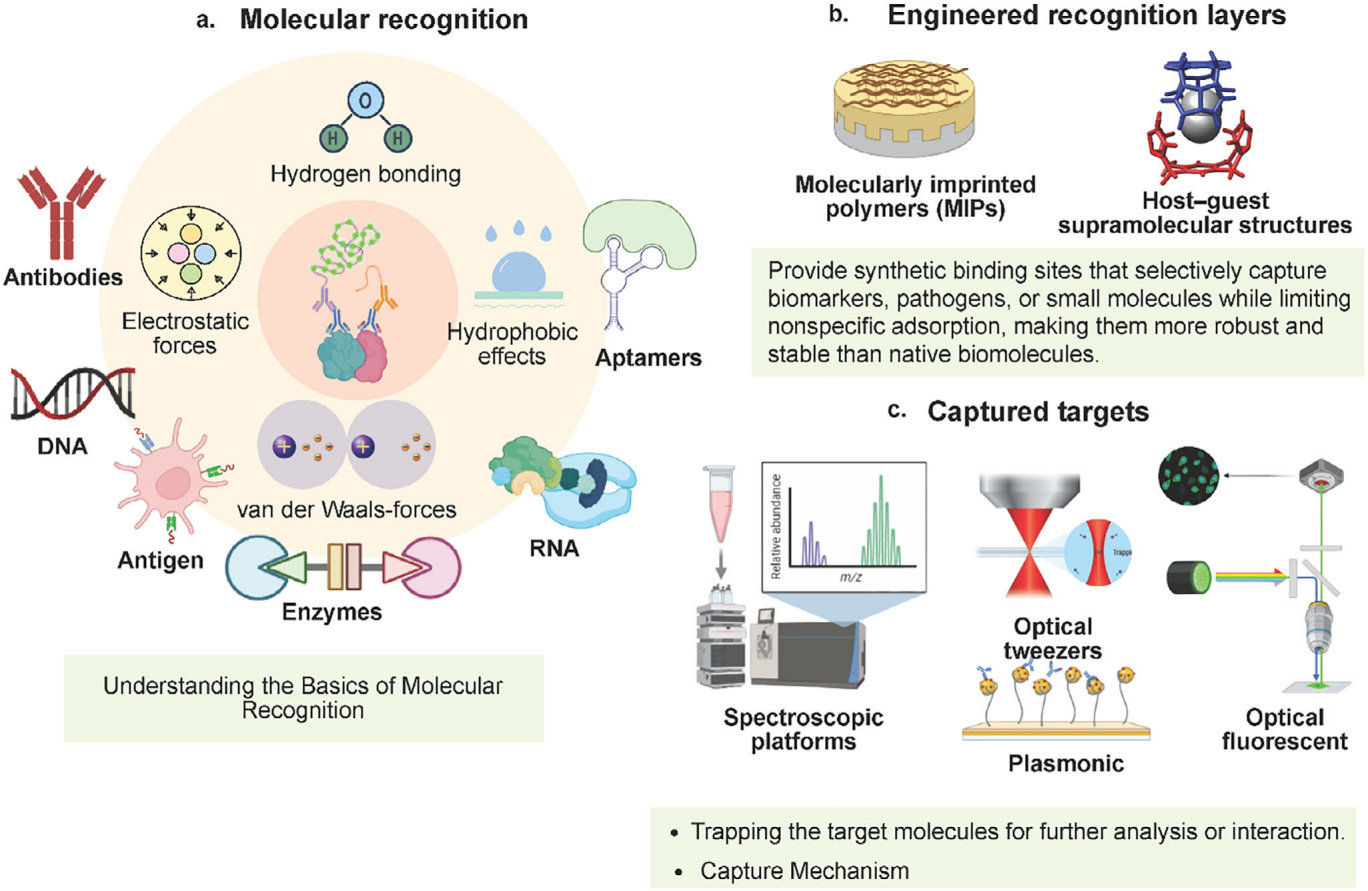
Molecular recognition and engineered capture architectures for selective biosensing. (a) Non‐covalent interactions enable recognition by antibodies, aptamers, enzymes, and nucleic acids. (b) Synthetic layers (MIPs and host–guest motifs) enhance robustness and selectivity. (c) Captured targets are analysed using spectroscopic, plasmonic, optical‐tweezer, and fluorescence readouts.

### 3.2 Signal‐Based Biosensing

Biosensing technologies convert biological or chemical interactions into electrical or optical signals that can be processed computationally. In neuromorphic molecular sensing, the distinctive step is converting these signals into asynchronous, event‐based representations. Experimental organic artificial neurons have shown concentration‐dependent spiking responses to ions and dopamine [56], while bioinspired electronic tongues have encoded ionic concentrations into spike trains [57]. Compared with conventional low‐power edge processing, the practical advantage of a spike‐based representation is therefore not low power alone, but the ability to exploit temporal sparsity: when informative molecular changes are intermittent, computation and communication can remain largely inactive between events, reducing redundant sampling, data movement, and memory access [58, 59]. This advantage is application‐dependent; slowly varying biomarkers sampled at low frequency may be handled as efficiently, or more simply by conventional edge processors. Native molecular‐to‐spike transduction also remains limited to a small number of chemical or biochemical targets. In addition to ion‐ and dopamine‐responsive artificial neurons, neurotransmitter‐responsive devices have reproduced integrate‐and‐fire chemical communication [60]. Together, these studies establish the feasibility of direct molecular or ionic event encoding, while highly multiplexed protein‐recognition arrays with native spike outputs remain a prospective capability.

For affinity‐based molecular sensing, the conversion from molecular concentration to a spike representation can be formulated through receptor‐binding dynamics. For a fractional receptor occupancy θ(t),

$$\;\frac{d\theta \left(t\right)}{dt}=k_{\mathrm{on}}\;C\left(t\right)\left[1-\theta \left(t\right)\right]-k_{\mathrm{off}}\theta \left(t\right),$$

where C(t) is the analyte concentration and $k_{\mathrm{on}}$ and $k_{\mathrm{off}}$ are the association and dissociation rate constants. At equilibrium,

$$\;\theta_{\mathrm{eq}}=\frac{C}{C+K_{D}}\;,\;\;K_{D}=\frac{k_{\mathrm{off}}}{k_{\mathrm{on}}}\;.$$

The resulting transducer signal, $S\;\left(t\right)=S_{0}+A\theta \left(t\right)$, could drive an integrate‐and‐fire element according to the following:

$$\;\frac{dV}{dt}=-V+\gamma \left[S\left(t\right)-S_{0}\right],$$

with a spike emitted when $V\geq V_{\mathrm{th}}$, followed by reset. In this representation, higher receptor occupancy can produce a higher mean spike frequency, providing a rate code for concentration [56, 57]. Binding kinetics can instead be retained in temporal features: during association, the observed rate constant is as follows:

$$\;k_{\mathrm{obs}}=k_{\mathrm{on}}\;C+k_{\mathrm{off}},$$

whereas after analyte removal the response decays according to $k_{\mathrm{off}}$. Faster signal accumulation therefore produces shorter first‐spike latency or shorter inter‐spike intervals, while association and dissociation can alternatively be represented by separate positive‐ and negative‐change events. Such combined rate–temporal encoding could preserve both concentration and kinetic information rather than reducing molecular dynamics to a single amplitude value [39, 56, 57]. The exact encoding function would require analyte‐specific calibration and remains a prospective design choice for multiplexed protein sensing.

Event thresholds should not be implemented as a single fixed amplitude criterion, because this could suppress gradual but biologically meaningful molecular changes. Instead, a multi‐timescale encoding strategy is envisaged. For a calibrated sensor signal (*S*(*t*)), rapid events could be generated when the deviation from a local baseline (*B*(*t*)) exceeds a noise‐scaled threshold,

$$\left[\left|S\left(t\right)-B\left(t\right)\right|>\lambda \sigma_{n}\left(t\right),\right]$$

where ($\sigma_{n}\left(t\right)$) represents the estimated local measurement noise and (λ) is selected according to the acceptable false‐event rate. In parallel, a slow pathway would integrate persistent subthreshold deviations over a longer interval, for example:

$$\left[G\;\left(t\right)=\int_{t-T}^{t}\left[S\left(\tau \right)-B\left(\tau \right)\right]\;,d\tau ,\right]$$

with a slow event generated when (|G(t)|) exceeds an analyte‐specific threshold. This allows small but sustained concentration changes to accumulate rather than being discarded. Positive and negative event channels can separately encode increasing and decreasing molecular signals, as demonstrated in asynchronous physiological spike encoders [61]. To prevent complete loss of slowly varying information, periodic low‐rate "checkpoint" measurements should also be retained irrespective of event generation. Importantly, (*B*(*t*)) should be corrected using nonresponsive reference or ratiometric channels rather than continuously fitted to the target signal itself, thereby reducing the risk that genuine slow biological changes are absorbed into baseline drift correction [62, 63]. Event thresholds should ultimately be analyte‐specific and linked to analytical variability and clinically meaningful changes in concentration rather than being defined solely by hardware sensitivity or data‐compression objectives [58].

### 3.3 Molecular‐Sensing Digital Twins for Diagnosis and Treatment Response Prediction

Patient‐specific digital twins are dynamic computational representations updated using longitudinal clinical, molecular, and pharmacological measurements to estimate evolving physiological states and treatment responses. Within the present framework, molecular sensing contributes selected time‐resolved biomarker inputs rather than continuous whole‐proteome measurements. Continuous or near‐real‐time protein sensing has been demonstrated for individual targets using affinity‐based and microfluidic platforms [33, 39, 44], whereas continuous proteome‐wide monitoring is not yet technically established. PBPK, PK/PD, and quantitative systems pharmacology models provide mechanistically structured implementations in which validated molecular measurements can update defined parameters and support predictions of drug exposure, response, and toxicity. Patient‐specific systems‐based digital twins have already been used to characterize dose–response relationships and predictive biomarkers in oncology [47].

Because metabolic pathways can be different for each person, standard dose schedules might not work for everyone. Proteomic measurements can inform selected PBPK parameters, but not the complete model. Quantitative abundance of drug‐metabolizing enzymes, such as CYP and UGT isoforms, can be used to scale enzyme‐specific metabolic intrinsic clearance when protein abundance has been linked to catalytic activity [64, 65]. Similarly, abundances of uptake and efflux transporters can inform transporter‐mediated clearance terms; targeted transporter proteomics has been incorporated into PBPK models to scale tissue uptake and efflux and to predict systemic and tissue statin exposure [66]. For circulating proteins, longitudinal measurements of albumin or α1‐acid glycoprotein may inform changes in the unbound drug fraction, ($f_{u}$), when drug‐specific binding constants are known [67]. However, most physiological PBPK parameters—including organ volume, tissue blood flow, glomerular filtration, membrane permeability, and drug‐specific kinetic constants—cannot be directly inferred from proteomics and must remain constrained by physiological measurements or independently validated models.

An important distinction is also required between tissue and circulating proteomics. Direct measurement of hepatic enzyme or transporter abundance generally requires tissue‐specific quantitative proteomics; consequently, conventional plasma protein panels should not be assumed to represent hepatic CYP or transporter abundance. Liquid‐biopsy approaches using circulating extracellular‐vesicle proteins have demonstrated the feasibility of estimating interindividual abundance of selected hepatic enzymes and transporters [64], but such relationships require protein‐specific validation before they can be used for patient‐specific PBPK updating. Thus, in the proposed architecture, longitudinal on‐chip proteomic measurements would update only PBPK parameters for which a validated mechanistic or empirical mapping from the measured protein to the corresponding model parameter has been established.

These principles are summarized in Figure 4, which illustrates a molecular‐sensing digital twin for clinical decision support and PBPK prediction. The framework combines molecular, clinical, and physiological data from patient‐specific sources, including wearable and implantable sensing devices, to dynamically update a mechanistic PBPK model. These devices serve as data‐acquisition or intervention interfaces rather than as PBPK compartments. The augmented digital twin supports pharmacokinetic and treatment‐response prediction, AI/ML integration, and individualized therapeutic decision‐making.

**FIGURE 4:**
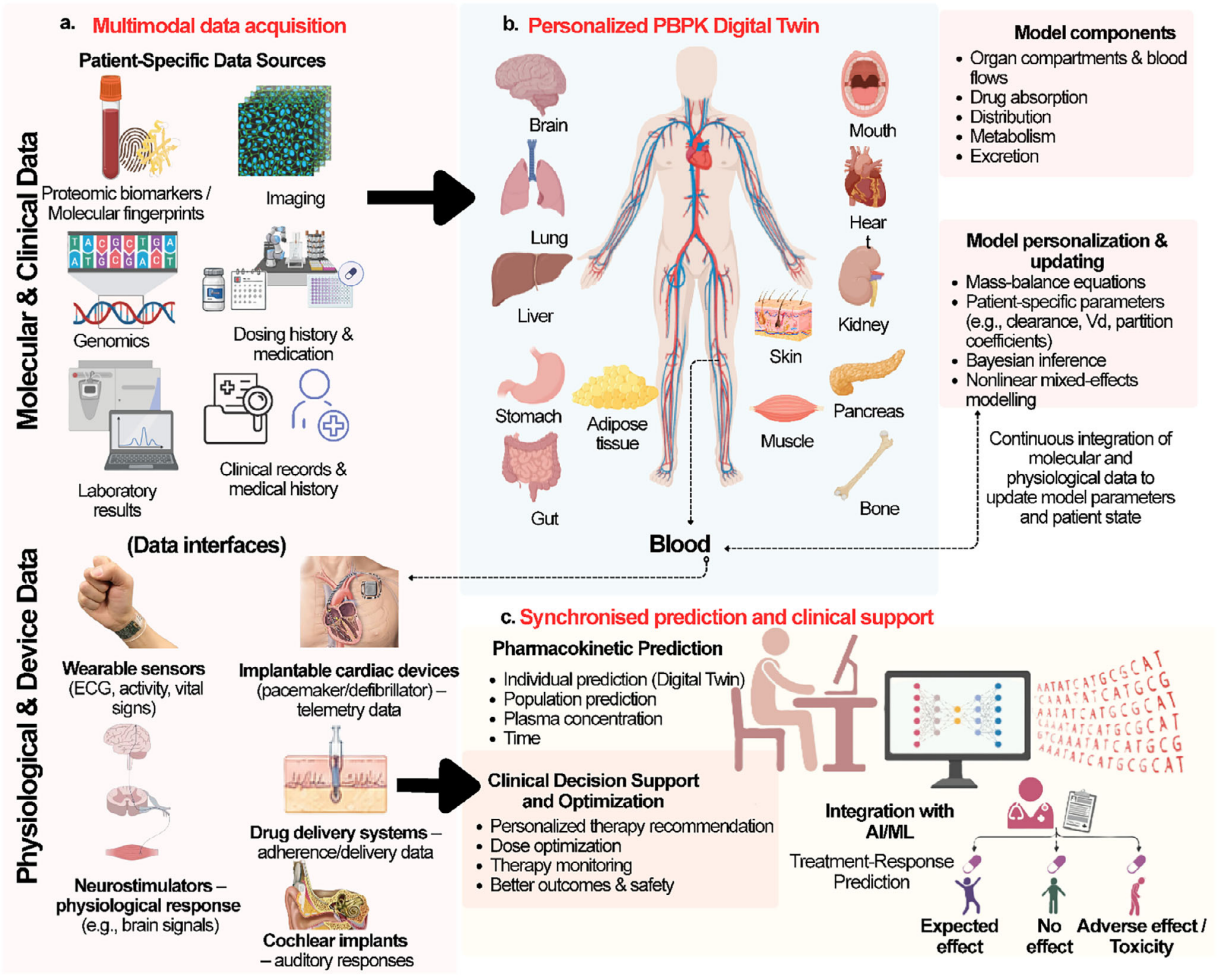
Patient‐specific physiologically based pharmacokinetic (PBPK) digital twin for pharmacokinetic prediction and clinical decision support. (a) Molecular, clinical, dosing, and physiological data streams. (b) Personalised PBPK model with inferred patient‐specific parameters. (c) Artificial intelligence/machine learning (AI/ML)‐enabled synchronization for treatment–response prediction and decision support. Devices serve as data‐acquisition interfaces.

Reliable digital twins require longitudinal integration of heterogeneous data sources together with explicit model updating, uncertainty management, and data provenance. Electronic health records, multi‐omics measurements, sensor streams, and laboratory data can provide complementary information, but their contribution must be governed by the intended model purpose and validated interfaces [68, 69, 70]. AI and machine‐learning methods can support pattern extraction and forecasting, but they do not replace mechanistic constraints or clinical validation [71, 72, 73]. Beyond clinical decision support, patient‐specific digital twins can support in silico drug development and regimen optimization. Mechanistic models have been used to study absorption, distribution, metabolism, and excretion and to investigate individualized pharmacological therapy [47, 74, 75]. Translation requires longitudinal integration of molecular, clinical, sensor, and treatment data together with traceable data provenance and interoperability across healthcare systems [69, 70].

### 3.4 Autonomous, Self‐Optimizing Biosystems

Autonomous and self‐optimizing biosystems extend sensing and prediction into closed‐loop therapeutic control. In such systems, physiological or molecular measurements are linked to feedback mechanisms that modify treatment within predefined constraints. Synthetic endocrine and biomarker‐responsive systems illustrate this principle, while stimuli‐responsive materials provide a route to controlled therapeutic release [76, 77, 78].

Clinical autonomy should nevertheless be graded rather than absolute. Early systems should operate as clinician‐supervised decision support, with autonomous adjustment restricted to prospectively validated operating ranges. Sensor‐quality failure, contradictory biomarker trajectories, high model uncertainty, or patient states outside the validated domain should suspend autonomous action and trigger clinical review. Flexible sensing and other bioelectronic interfaces may support real‐time feedback, but their value depends on robust sensing, control algorithms, and patient‐specific safety constraints [77, 79, 80].

Accordingly, the defining issue is not simply whether AI is connected to a biosensor, but whether sensing, interpretation, and actuation are integrated within a validated feedback architecture. Representative concepts include biomarker‐driven therapeutic systems and autonomous microrobotic delivery platforms [78, 81]. Table 1 summarizes the broader distinctions between traditional biosensors and on‐chip intelligent systems.

**TABLE 1:** On‐chip intelligence systems compared with traditional biosensors.

Feature	Traditional biosensors	On‐chip intelligence	Reference
Materials	Enzymes, polymers, and gold enhance the detection system's specificity and sensitivity.	Advanced nanomaterials, including TMDs and nanozymes, can enhance transduction, sensitivity, and device integration.	[53, 82]
Wearable sensors	Applications that require real‐time data processing are overly large and require extra data processing devices.	Blend discrete, flexible electronics into normal clothing for real‐time monitoring.	[79, 83]
Molecular capture	The interaction is passive, which can be time‐consuming.	Regenerable or active recognition interfaces can be coupled with adaptive local inference and calibration.	[34, 39, 84]
Edge processing: An on‐chip AI model	Diagnosis is delayed when data are centralized and analyzed externally.	On‐chip processing enables real‐time data analysis and sensor response.	[1, 58, 85]
AI role	Operator‐dependent data augmentation and basic signal processing.	Reduce noise, extract features, and apply predictive analytics with algorithms to enhance accuracy and enable real‐time decision‐making.	[13, 84, 86, 87, 88]
Signal conversion	Fixed transduction with conventional downstream signal processing.	Converts and locally interprets electrical, optical, or event‐based signals for adaptive diagnostics.	[21, 56, 57, 89]
Twin synchronization	Traditional biosensors cannot synchronize with various devices in real time.	Integrates longitudinal sensor and clinical data within a patient‐specific computational model for dynamic monitoring and prediction.	[69, 70]
Predictive autonomous action	Responses that require human intervention for continuation.	AI‐based proactive health management and individualized care based on real‐time data.	[77, 78, 90, 91]

## 4 Current Challenges and Future Directions

Translating biomimetic intelligence from an appealing concept to a clinically credible platform will require co‐optimizing molecular recognition, device reliability, and inference. At the molecular interface level, a primary bottleneck maintains specificity at scale. As multiplexing expands, cross‐reactivity, competitive binding, and matrix effects accumulate, and even strong host–guest chemistries and biomolecular receptors can drift under ionic‐strength, pH, and fouling variations typical of real biofluids [35, 36, 50]. Near‐term progress should prioritize (i) regenerable recognition layers and antifouling interfaces, (ii) redundant/orthogonal sensing (multiple probes per analyte and multimodal readouts), and (iii) embedded reference channels and orthogonal calibration controls to separate binding drift from true biological change [50, 62, 63]. Just as importantly, recognition performance should be reported in standardized terms (selectivity in serum, regeneration cycles, calibration stability), not solely in buffered benchmarks. Although application‐specific validation will ultimately be required, useful engineering targets for future continuous on‐chip systems could include baseline drift of ≤ 5% over 24 h, fouling‐induced signal loss of ≤ 10% after 24 h exposure to physiologically relevant biofluids, cross‐reactivity of ≤ 5% toward major interferents, and inter‐device variation of ≤ 10%. For regenerable recognition interfaces, retention of ≥ 90% of the initial response after at least 10 regeneration cycles would provide an additional practical benchmark. These targets are broadly consistent with performance already approached by advanced antifouling and affinity‐based biosensing platforms. At the acquisition and computation level, continuous sampling must also operate under tight energy and telemetry budgets. Importantly, current continuous molecular sensors generally monitor individual analytes or limited biomarker panels rather than the proteome as a whole. Extending these platforms toward multiplexed longitudinal proteomic monitoring will require parallel recognition channels, reversible sensing interfaces, robust antifouling, and long‐term calibration stability [33, 39, 44]. A further design requirement arises from the extreme concentration range of the plasma proteome, which spans more than 10 orders of magnitude [92]. The proposed platform, therefore, would not rely on a single uniform sensing range, but on parallel microfluidic channels matched to different concentration regimes. High‐abundance targets could be directed through diluted streams or interrogated using lower‐affinity recognition elements, whereas low‐abundance proteins could be measured using higher‐affinity capture and, where necessary, on‐chip enrichment. Selective removal of dominant proteins may additionally be incorporated upstream for specific low‐abundance panels, although such depletion should be used cautiously because co‐depletion can distort the native protein distribution; integrated microfluidic depletion of highly abundant serum proteins has nevertheless been demonstrated experimentally [93, 94]. Nonspecific adsorption and progressive fouling would be mitigated using antifouling coatings or protective membranes together with nonbinding reference channels and differential calibration [50]. Finally, longitudinal monitoring requires reversible recognition: receptor occupancy must recover sufficiently rapidly to follow changes in analyte concentration. This may be achieved through recognition elements with appropriate association/dissociation kinetics or periodic active regeneration; active‐reset protein sensors have demonstrated rapid regeneration and continuous in vivo protein monitoring. Regeneration should be sufficiently strong to dissociate the target but sufficiently mild to preserve receptor conformation, surface attachment, and binding accessibility [39]. Stable immobilization layers and antifouling protection are therefore important for limiting receptor loss and nonspecific surface changes during repeated cycles [50, 95]. Baseline stability can additionally be maintained using nonbinding reference channels or ratiometric/differential readouts that distinguish common‐mode drift from target‐dependent responses [62]. After each regeneration cycle, recovery of the pre‐binding baseline together with the calibration slope or apparent affinity should be monitored; progressive changes beyond predefined limits would trigger recalibration or exclusion of that sensing channel. Long‐term operation should additionally demonstrate recovery of baseline and calibration characteristics while meeting the regeneration‐retention benchmark defined above. Thus, sample conditioning, affinity matching, antifouling protection, and sensor regeneration are treated as integrated components of the proposed architecture rather than independent corrections. High‐rate biosensing is often information‐inefficient, most samples are redundant, yet power and bandwidth are consumed continuously. Event‐driven acquisition and near‐/in‐sensor computation therefore become central design goals, especially when paired with asynchronous neuromorphic primitives that compress sparse, transient molecular events [58, 59, 96, 97]. Recent organic bioelectronic work illustrates feasible paths toward low‐energy, high‐frequency event‐based sensing and closed‐loop actuation [98], while flexible in‐sensor computing circuits based on organic electrochemical transistors highlight manufacturable routes to embedding computation at the sensor edge [99]. However, clinical translation will be limited by reliability (drift, hysteresis, regeneration limits), packaging/biocompatibility, and rigorous acceptance testing under realistic operating cycles [77, 80, 95].

Progression from molecular monitoring to autonomous therapeutic control requires staged validation. Verification and analytical validation should first establish accuracy, precision, analytical sensitivity and range, selectivity and interference, response time, drift, regeneration stability, cross‐channel effects, and device‐to‐device reproducibility under intended‐use conditions [100]. The inference and control algorithms should then undergo independent external validation for calibration, robustness to missing or noisy inputs, uncertainty estimation, subgroup performance, and predefined failure modes. Prospective silent deployment should assess the integrated system in the intended clinical environment before supervised studies in which recommendations remain subject to clinician approval [101]. Advancement to autonomous control would require prospective, preferably multicenter randomized trials demonstrating clinical benefit or prespecified non‐inferiority and acceptable safety relative to standard care; automated insulin‐delivery trials provide an established precedent.

Runtime safety requires layered gates that prevent an isolated sensing or modelling error from directly producing a therapeutic action. When clinically appropriate, putative molecular events should be confirmed through temporal persistence, agreement between redundant recognition elements, or an orthogonal measurement. Embedded reference channels and calibration controls should assess baseline stability and sensor integrity; measurements that exceed validated drift, calibration, cross‐channel, or signal‐quality limits should be excluded from autonomous control [62]. Model outputs should likewise include calibrated uncertainty estimates, and high‐uncertainty or out‐of‐distribution patient states should trigger fallback to a predefined safe mode rather than treatment escalation [102]. Clinician oversight should remain hierarchical: early therapeutic changes require human approval, while later bounded low‐risk adjustments may operate automatically only within prospectively validated limits; contradictory measurements, repeated calibration failures, unexpected trajectories, or high model uncertainty should be escalated for clinical review [101, 103]. All sensing inputs, model outputs, therapeutic actions, overrides, and failure events should be logged for audit and post‐deployment surveillance.

At the longitudinal modelling level, generalizable molecular trajectories require harmonized protocols and representative longitudinal resources. Large‐scale longitudinal proteomics is now resolving age‐ and disease‐associated trajectories with clinically meaningful follow‐up [104, 105, 106], but cohort composition, pre‐analytics and platform differences can still dominate learned signatures and impede transportability. A pragmatic direction is to define benchmark panels and minimum metadata (pre‐analytic variables, calibration/QC traces, assay versioning), align continuous sensor outputs with orthogonal reference assays at scheduled intervals, and establish cross‐site proficiency testing so that models can be updated without eroding interpretability. Finally, actionable digital twins require causal validity, explicit uncertainty quantification, and auditable failure modes [42, 102, 107, 108, 109, 110]. Longitudinal association alone would not be interpreted as evidence of a causal physiological relationship. Instead, the proposed digital twin would represent candidate relationships within a mechanistically constrained structural causal model, for example:

$$\;z_{t+1}=F\left(z_{t},a_{t},c_{t},\varepsilon_{t}\right)$$

Here, causal decoding refers to the identification of candidate directional relationships from time‐resolved molecular data rather than proof of causation itself [42]. Counterfactual validity refers to whether the model can correctly predict the outcome expected under a specified intervention that differs from the observed course. Because the unobserved counterfactual for an individual cannot be measured directly, such validity must be assessed using independent intervention data, appropriately designed observational comparisons such as target‐trial emulation, or consistency across different experimental or physiological conditions [108, 109]. A control node is therefore not simply a highly connected or strongly predictive biomarker, but a variable whose perturbation is predicted to move defined downstream physiological targets toward another state [43]. For example, if longitudinal data show that protein (X) predicts progression toward disease state (Y), (X) would initially remain an associative candidate. Stronger causal support would require appropriate confounder adjustment and evidence that experimentally or clinically altering (X) produces the predicted change in (Y). Only relationships supported by such validation would be considered for control‐oriented digital‐twin decisions.

Where $z_{t}$ denotes the physiological state, $a_{t}$ a measured intervention or exposure, $c_{t}$ observed time‐varying confounders, and $\varepsilon_{t}$ unresolved influences. Time‐series causal‐discovery methods may identify candidate directed dependencies from longitudinal molecular measurements [42], but these relationships would require additional causal evaluation. When treatment or exposure histories are available, longitudinal causal methods such as marginal structural models can account for measured time‐dependent confounding [110], while observational treatment comparisons can be formulated as explicit target‐trial emulations [108]. The relevant quantity is therefore an interventional contrast, such as,

$$E\left[Y_{t+h}|do\left(\;A_{t}=a\right)\right]-E\left[Y_{t+h}|do\left(\;A_{t}=a^{\prime }\right)\right],$$

rather than the corresponding observational association $E\left[Y|A=a\right]-E\left[Y|A=a^{\prime }\right]$. Candidate relationships should additionally be tested for stability across patients, cohorts, physiological conditions, or controlled perturbations, since invariance across changing environments provides further evidence for causal structure [109]. Where feasible, intervention data from treatment changes, controlled experimental systems, or microphysiological models would provide stronger validation. Relationships supported only by observational prediction would remain labeled as associative, would contribute to forecasting, but would not be treated as validated control links for autonomous therapeutic decisions. Their clinical use also requires a clearly defined intended use, traceable data provenance, and appropriate model risk management [68, 69, 70]. Given these causal and governance constraints, near‐term implementation may therefore favor a "minimal viable twin," defined here as a disease‐ and decision‐specific patient model that (i) uses a limited set of validated inputs, including longitudinal molecular measurements and relevant clinical or pharmacokinetic data; (ii) contains a mechanistic PK/PD or QSP core linking these inputs to a predefined outcome; (iii) is updated as new patient measurements become available; and (iv) reports prediction uncertainty for a single specified clinical decision. Such a twin need not reproduce the complete patient or entire proteome; broader proteomic profiling may identify relevant molecular signatures, while the deployed twin can use the validated subset required for the intended decision. A realistic initial application is treatment‐response and dose optimization in B‐cell non‐Hodgkin lymphoma treated with CD20×CD3 bispecific antibodies. Patient‐specific digital twins have already been generated for mosunetuzumab‐treated NHL patients using individual pharmacokinetic, tumor‐size, and biomarker data to characterize dose/exposure‐response and biological determinants of response [47]. Related oncology digital twins have reproduced patient‐specific cellular kinetics following engineered T‐cell therapy [111], while on‐chip affinity sensing has demonstrated time‐resolved measurement of protein responses to anticancer treatment [44]. Thus, an initial implementation could combine serial molecular measurements with pharmacokinetic and clinical response data to update a focused treatment‐response twin, with autonomous treatment adjustment reserved for later prospective validation.

## 5 Application of Biomimetic Intelligence in Diagnostics and Precision Medicine

The preceding sections established the sensing, computational, and digital‐twin components of the proposed framework; this section focuses on representative diagnostic and precision‐medicine applications. Biomolecular recognition and self‐organization remain relevant biological principles [36, 112], while patient‐specific digital twins can integrate molecular and clinical information to track disease progression and treatment response. Their coupling with microphysiological systems, including organ‐on‐chip platforms, provides an experimentally informed route for treatment monitoring and pharmacokinetic investigation [113, 114]. Several individual elements of the proposed design have been demonstrated experimentally in integrated or modular on‐chip systems. Organ‐on‐chip and microfluidic systems have been integrated with multi‐omics and proteomic analyses, including mass spectrometry and data‐independent acquisition (DIA‐MS), thereby demonstrating viable pathways from on‐chip biological models and sample processing to proteomic assessments [115, 116, 117]. Microfluidic systems for single‐cell proteomics, which encompass integrated workflows for sample collection and preparation suitable for subsequent proteomic analysis, demonstrate that significant segments of the suggested molecular‐profiling layer can be executed on‐chip. Spatial and multiplexed molecular imaging, alongside electrical, mechanical, and imaging‐based functional sensing, have been integrated into organ‐on‐chip and microfluidic processes [115], establishing experimental foundations for integrating molecular and functional assessments within chip‐based frameworks. Simultaneously, AI and machine learning methodologies have been used to interpret and integrate multi‐omics data generated by organ‐on‐chip and similar platforms, thereby enhancing the computational feasibility of sophisticated analyses [118, 119]. Figure 5 provides a broad overview of representative applications. Nonetheless, the comprehensive integration, specifically adaptive molecular sensing, longitudinal targeted proteome profiling, event‐driven processing, autonomous adaptation, and patient‐specific digital‐twin updating within a single, continuously operating platform. Specifically, ongoing real‐time proteome analysis with dynamic on‐chip state modifications and feedback‐driven autonomous decision‐making remains a prospective developmental goal. Thus, the current architecture ought to be perceived as a synthesis and a proposed integration pathway based on experimentally validated modules, rather than as an assertion that the entire end‐to‐end system has been experimentally demonstrated.

**FIGURE 5:**
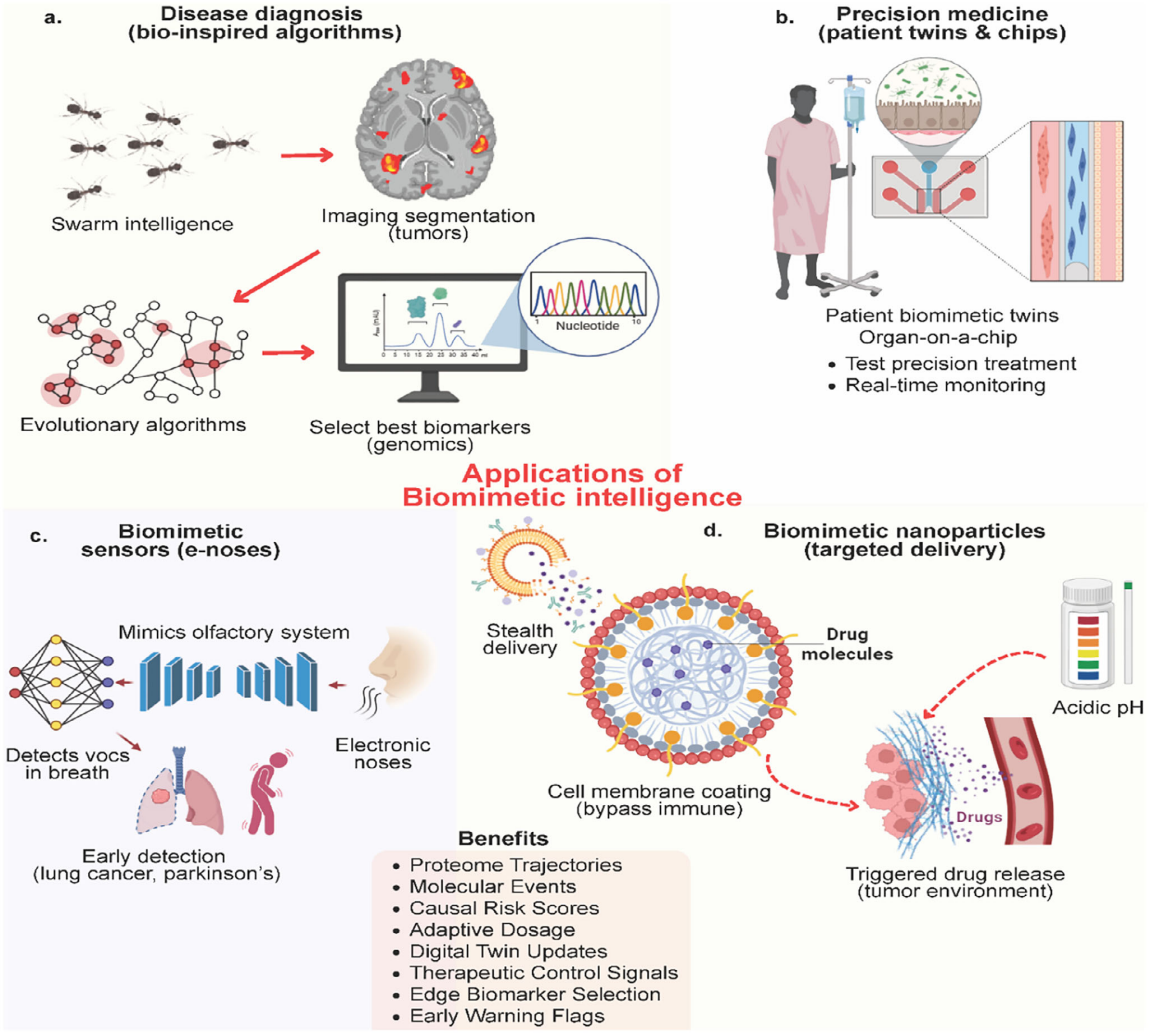
Applications of biomimetic intelligence in healthcare that integrate biological principles of sensing, collective information processing, physiological adaptation, molecular recognition, and targeted interaction. (a) Disease diagnosis using bio‐inspired swarm and evolutionary algorithms supports imaging segmentation and the selection of informative biomarkers from genomic data. (b) Patient twins and organ‐on‐a‐chip platforms are used to simulate relevant physiological conditions for customized treatment testing and real‐time therapeutic monitoring. (c) Bio‐inspired sensor arrays mimic aspects of the olfactory system to detect volatile molecular patterns associated with diseases such as lung cancer and Parkinson's disease. (d) Cell‐membrane coatings are used to provide immune evasion and biological compatibility, while disease‐associated environmental cues, such as acidic tumour microenvironments, can be exploited to trigger localized drug release. Across these applications, biomimetic principles are translated into sensing, inference, prediction, adaptation, and intervention.

## 6 Conclusion

On‐chip biomimetic intelligence provides a framework for moving molecular biosensing from static detection toward longitudinal, adaptive interpretation. Its practical development will require the integration of robust molecular recognition and transduction, event‐efficient local processing, longitudinal protein‐panel monitoring, and mechanistically constrained patient‐specific models. Near‐term progress depends on regenerable, antifouling sensing interfaces; embedded calibration; harmonized longitudinal datasets; explicit uncertainty quantification; and causal validation of model relationships. Therapeutic autonomy should remain bound by validated operating limits and clinician oversight. Rather than attempting to reproduce the complete proteome or the patient, initial translation should focus on disease‐ and decision‐specific minimal viable twins that combine validated molecular and clinical measurements to meet a defined clinical objective. Demonstrating reproducibility, cross‐site transportability, and prospective clinical utility will be the critical next step toward clinically credible biomimetic‐intelligence systems.

## Author Contributions

**Bakr Ahmed Taha**: conceptualization and methodology, writing – original draft, and writing – review and editing. **Ali J. Addie**: writing – original draft and writing – review and editing. **Khansaa Basem Fadhil**: writing – review and editing. **Adawiya J. Haider**: writing – review and editing. **Ahmed Jamal Jasim**: writing – review and editing. **Norhana Arsad**: writing – review and editing. All authors have reviewed and accepted the published version of the article.

## Conflicts of Interest

The authors declare no conflicts of interest.

## Data Availability

Data sharing is not applicable to this article as no datasets were generated or analyzed during the current study.
